# Thymol-Functionalized Hyaluronic Acid as Promising Preservative Biomaterial for the Inhibition of *Candida albicans* Biofilm Formation

**DOI:** 10.1021/acsmacrolett.3c00208

**Published:** 2023-07-18

**Authors:** Elisa Sturabotti, Vyali Georgian Moldoveanu, Alessandro Camilli, Andrea Martinelli, Giovanna Simonetti, Alessio Valletta, Ilaria Serangeli, Alessandro Giustini, Elena Miranda, Luisa Maria Migneco, Fabrizio Vetica, Francesca Leonelli

**Affiliations:** † Department of Chemistry, Sapienza University of Rome, Piazzale Aldo Moro 5, 00185 Rome, Italy; ‡ Department of Environmental Biology, Sapienza University of Rome, Piazzale Aldo Moro 5, 00185 Rome, Italy; § Department of Biology and Biotechnologies “Charles Darwin”, Sapienza University of Rome, Piazzale Aldo Moro 5, 00185 Rome, Italy

## Abstract

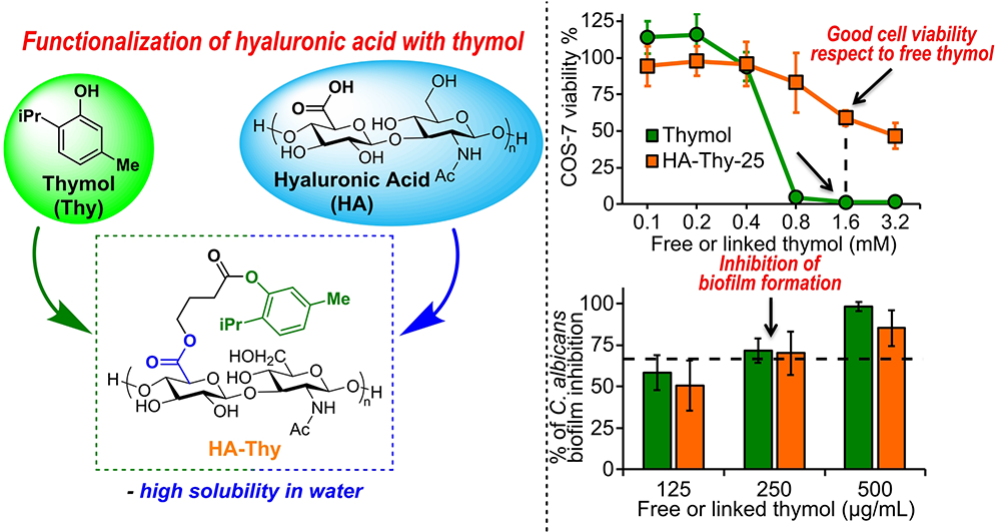

Hyaluronic acid (HA) is a naturally occurring biopolymer that has been employed for a plethora of medicinal applications. Nevertheless, as HA is a natural polysaccharide, it can be a substrate able to promote microbial growth and proliferation. Biopolymer–drug conjugates have gained attention over the years to overcome drawbacks of each single component. Within this context, thymol (Thy), a phenolic compound occurring in essential oils (EOs) extracted from *Thymus* and *Origanum*, has been largely studied for its antimycotic applications. However, it is characterized by a low water solubility and moderate cytotoxicity. Herein, we report an innovative HA–thymol conjugate (HA-Thy) biomaterial to circumvent the drawbacks of free thymol use by providing the polymer conjugate with the beneficial properties of both components. Preliminary biological tests evidenced the decrease of thymol cytotoxicity for the HA-Thy conjugate, paired with a promising antibiofilm formation activity against *Candida albicans*, similar to pure thymol, highlighting its potential application as a preservative biomaterial in formulations.

Hyaluronic acid (HA) is a multipurpose biomaterial with a wide variety of biological activities, widely used in both cosmetic and medicinal formulations.^1−6^ From a structural point of view, it is a negatively charged linear polysaccharide composed of a disaccharide unit of d-glucuronic acid and *N*-acetyl-d-glucosamine linked by β-(1,4) and β-(1,3) glycosidic bonds. Interestingly, its physicochemical as well as biological properties can be easily tuned by chemical modifications and conjugation to other molecules. Indeed, due to its features and biocompatibility, HA has been employed as a polymeric support for drug conjugates made to deliver active principles and for bioactive polymeric materials.^7−10^

Essential oils (EOs) are a wide class of naturally occurring molecular structures that have attracted the attention of the scientific community over the years due to their extensive and various biological activities. Among the vast realm of EOs, thymol (2-isopropyl-5-methyl phenol, Thy) is a natural hydrophobic phenol monoterpene found in essential oils derived from *Thymus*, *Origanum*, and *Coridothymus* that shows antibacterial,^11^ anti-inflamatory,^12^ antifungal,^13^ and antitumor^14^ activities, among others. When dealing with such a large variety of biological activities, the selective targeting of active principles remains a key challenge for medicinal applications. Moreover, the use of thymol and EOs, in general, for biological applications is hindered by its low solubility in water^15^ and cytotoxic effects toward both healthy and pathological cells.^16,17^ For these reasons, the scientific community has focused its efforts over the selection of suitable conjugation methodology of the EOs, or of pure components, to low molecular weight molecules^18^ or to their encapsulation into 2D and 3D polymeric matrices.^19^ The physical loading of Thy into chitosan-based nanogels^20^ or liposomes^21^ and cellulose nanoparticles^22^ ensures the persistency of its antibacterial and antimycotic properties against *Staphylococcus aureus*, *Acinetobacter baumanii*, and *Pseudomonas aeruginosa* or *Candida* species, providing, at the same time, low cytotoxic effects of the drug carriers on human cells. Also, HA, in the form of liposomes, gels, or electrospun fibers, has been employed as matrices for conventional antimycotic drugs, allowing the hydrophobic molecules to improve their bioavailability by encapsulation.^23^ Due to healthcare attention and improvement and massive or wrong medicinal usage, many microorganisms have developed drug resistance, increasing their lower limit of susceptibility to common drugs. This phenomenon is known worldwide as drug resistance. Among the species belonging to the genus *Candida*, *Candida albicans* is the species that most frequently causes disease and that constitutes one of the principal causes of nosocomial infections.^24^*C. albicans* expresses strong resistivity to conventional antifungal drugs, such as fluconazole and amphotericin B, and, unfortunately, the formation of resistant biofilms exacerbates its acuteness compared to planktonic cells.^25^ Since the *Candida* biofilm are usually found in prosthetic devices, catheters, and cardiac devices, the treatment of biofilm infections is still challenging.^26^ Thereby, the urgency for the development of a system able to eradicate eukaryotic or prokaryotic microorganism biofilms represents one of the main challenges of this century of biomedicine.

Within this context and taking advantage of our group expertise in the synthesis of bioactive organic compounds, biomaterials, molecular carriers for active principles delivery and polymer chemistry,^27−33^ we envisioned the possibility of developing a facile strategy for the grafting of thymol onto HA (Figure 1). In this way, by merging the key polymeric features of HA with the biological activity of Thy, it is possible to simultaneously tackle the main issues of solubility and toxicity of the phenol monoterpene. Moreover, if the peculiar physicochemical and biological properties of both ingredients are maintained, the HA–thymol conjugates could represent an innovative and unprecedented ingredient for biomedical formulations with, for instance, preservative activity.^34^ Herein, our results on the synthesis and physicochemical properties of an innovative thymol-functionalized HA biomaterial are presented, highlighting how the functionalization not only drastically reduces the cytotoxicity of thymol, but also furnishes potential antifungal activity to HA.

**Figure 1:**
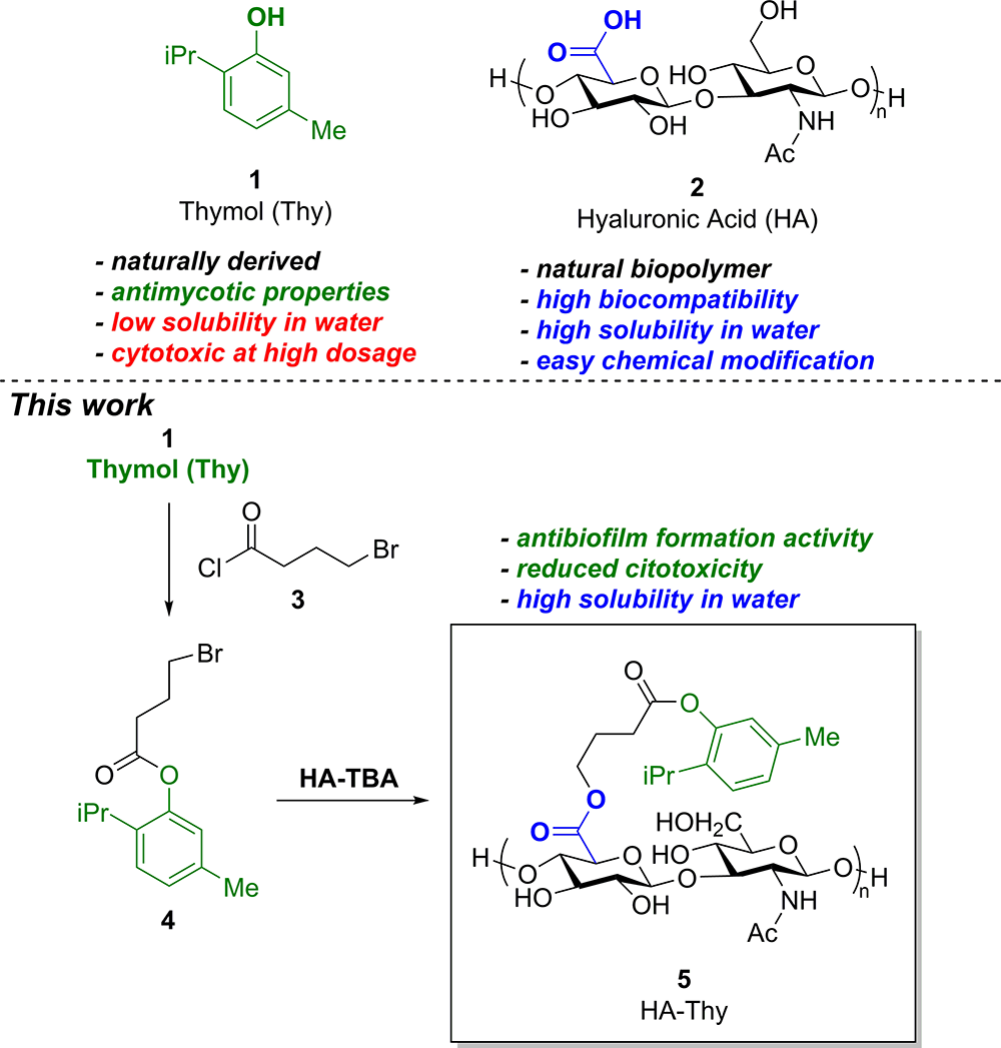
Structures of the natural essential oil thymol (Thy, **1**), hyaluronic acid (HA, **2**), 4-bromobutyryl chloride (linker, **3**), thymol ester (**4**), and the biomaterial HA-Thy (**5**) synthesized in this work.

Our investigation started with the identification of a suitable linker for the conjugation of thymol (**1**) and HA (**2**). Based on our previous works,^8^ we envisioned the possibility of esterifying the thymol–OH group with 4-bromobutanoyl chloride (**3**). In fact, the so-obtained thymol ester with the terminal Br substitution (**4**) could react with a tetrabutylammonium hyaluronate (HA-TBA) salt, simply prepared from commercially available HA, in a nucleophilic substitution, yielding the diester HA-Thy (**5**). The advantage of using such a linker resides in the presence of labile ester functionalities that are practical, easy to insert, and also susceptible to hydrolysis in biological media, allowing for a possible release of the unmodified components of the bioconjugate over time.

Thus, compound **4** was efficiently prepared by simple esterification between **1** and **3**, yielding 94% of compound **4** (^1^H and ^13^C NMR and ATR-FTIR spectra in Figures S1, S2, and S3, respectively). HA-TBA, prepared following a reported procedure^35^ from commercially available HA (1000–1500 kDa), was reacted in DMSO at room temperature with compound **4** for 5 days, using two different **4**/HA-TBA ratios, 0.25:1 and 0.50:1. The chosen ratios of reactants were carefully selected to ensure a partial functionalization of HA-COOH moieties, hence obtaining water-soluble HA derivatives (concentration between 2% and 0.5 wt %/v for homogeneous polymeric solutions). Crude **5** samples, isolated by precipitation in brine/EtOH directly from the reaction mixture, followed by centrifugation, were subsequently purified via extensive dialysis (14 kDa MWCO) for 5 days. The two HA-Thy derivatives, obtained with the two different reagent ratios, gave cotton-like material after freeze-drying, and they were completely characterized by FTIR, UV, and ^1^H NMR analyses. Compared to pristine HA, the FTIR spectra of the obtained samples highlighted the presence of a new distinctive broad band of ester functionalities at ca. 1740 cm^–1^, proving the formation of new ester moieties in the HA-Thy derivatives (Figure S4). A qualitative analysis of the incorporation of thymol on HA was performed by UV–vis spectroscopy, which evidenced the presence of a distinctive band at 264 nm, ascribable to the thymol aromatic ring, blue-shifted and broadened with respect to that of pure thymol (276 nm) due to the esterification of the phenolic OH group and to the conjugation to the polymeric backbone (Figure S5). This result was further confirmed by ^1^H NMR analysis, which allowed the quantification of the degree of substitution via integration of the characteristic peaks at 2.00 ppm, related to the acetylic group in HA and between 6.90 and 7.40 ppm of thymol phenyl groups. These studies highlighted that by using reagent ratios of 0.25:1 and 0.50:1, the obtained degrees of substitution of HA-Thy were ca. 25% (HA-Thy-25, **5a**) and 50% (HA-Thy-50, **5b**), respectively (Figures S6, S7, and S8). Preliminary semiquantitative tests showed that the presence of a higher concentration of lipophilic thymol moieties bonded to HA reduces the water solubility of **5b** (less than 0.5 wt %/v; [HA-Thy-50] = 10 mM, containing 5 mM of bonded thymol at 25 °C; free thymol solubilizes in water at 5.7 mM at the same temperature),^36^ dramatically hindering the bioapplications of the conjugate. Due to these findings, we decided to proceed with the bioactivity studies only on HA-Thy-25 (solubility up to 1.5 wt %/v; [HA-Thy-25] = 33 mM containing 8.3 mM bonded thymol at 25 °C).

As mentioned previously, the nonselective cytotoxic effect of thymol poses a significant challenge to its use as an antimycotic agent.^16,17^ To explore the potential application of the newly prepared HA-Thy-25 bioconjugate as an antimicrobial material, we conducted a cytotoxicity test on COS-7 cells, a fibroblast-like cell line derived from monkey kidney tissue, using the MTT (3-(4,5-dimethylthiazol-2-yl)-2,5-diphenyltetrazolium bromide) assay to assess cell viability (cells density: 100000 per well). In this assay, the MTT salt is reduced to purple formazan crystals by mitochondrial dehydrogenase, which is active in healthy cells. The amount of formazan produced is proportional to the number of cells in the culture and could be measured spectrophotometrically. The COS-7 cells were treated with varying concentrations of **5a**, ranging from 0.4 to 12.8 mM, for 48 h. Free thymol and pristine HA were used as comparisons at concentrations adjusted to the amount of each moiety in **5a**. For instance, 1 mL of a 12.8 mM solution of HA-Thy-25 (500 μg/mL) contained 3.2 μmol of thymol. Thus, a parallel experiment was conducted starting from a 3.2 mM solution of pure thymol. The 1:2 serial dilutions of the Thy solution were made for all the following experimental points. Additionally, another control sample was carried out with 1:2 dilutions from a 12.8 mM solution of pristine HA, since the molecular weights of HA and HA-Thy are essentially similar (Table S2). The concentrations of **5a** selected for the treatment were chosen to test the active concentration of free thymol against *C. albicans* (*vide infra*).^37^ As indicated in the Methods section (Supporting Information), the compounds were dissolved at room temperature and in the dark for 24 h before the experiment. To determine whether the incubation of the medium at r.t. could result in the deterioration of the medium components, the result of a MTT experiment conducted using the medium left at r.t. for 24 h was compared to that obtained with a medium kept at 4 °C. The findings demonstrated that this incubation did not affect the viability of the COS-7 cells (data not shown).

The graph in Figure 2 shows the result of the MTT viability test in COS-7 cells treated with free thymol, HA-Thy-25, and HA alone. Values are normalized to the viability of untreated control COS-7 cells. As expected, free thymol caused a striking toxicity at the highest concentrations (0.8, 1.6, and 3.2 mM), which diminished with decreasing concentration, as assessed also by García-Salinas at al.^38^ Instead, the cell viability in the presence of HA-Thy-25 was comparable to that of cells treated with HA at all tested concentrations. The high variability of the results obtained from the HA sample was likely due to its high viscosity, resulting in the variable recovery of dissolved formazan crystals for that group of samples.

**Figure 2:**
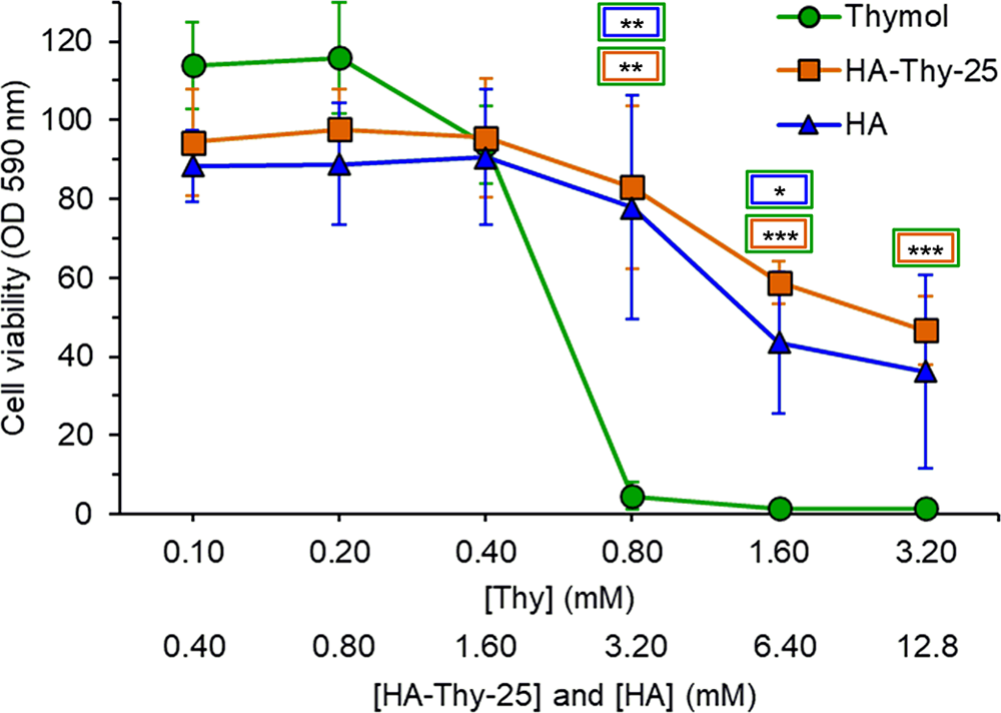
Cell viability MTT assay of cultured cells treated with free thymol (green), HA-Thy-25 (orange), and HA (blue) for 48 h. The concentrations of free thymol, HA-Thy-25, and HA are reported on the *x*-axis and are explained in the main text. All experiments were performed at least 3 times. Statistical test used: Students’ *t* test; *p* value: *p ≤ 0.01; **p ≤ 0.002; ***p ≤ 0.0009.

Prompted by these excellent results and considering that thymol could interfere with *C. albicans* biofilm formation, as well as acting on mature biofilms,^37^ we tested the plausible antifungal properties and biofilm inhibition of **5a** against *C. albicans* ATCC 10231. The antimicrobial activity of HA-Thy-25 and thymol was studied by comparing the same amount of free **1** and conjugated thymol in **5a**. Hyaluronic acid activity was investigated at concentrations equal to that of HA-Thy-25. Similarly to the cytotoxicity measurements, all the compounds were dissolved directly in the microorganism growth medium (RPMI 1640 medium) at low temperature and in the dark for 24 h. All compounds were then diluted 1:2 for each data point. The minimum inhibition concentration at 48 h (MIC) was tested in a range of thymol concentration between 0.977 and 500 μg/mL (corresponding to 0.006 and 3.2 mM). The minimum biofilm inhibition concentration (BMIC) was instead evaluated at 125, 250, and 500 μg/mL of thymol (0.8, 1.6, and 3.2 mM) after 48 h of experiments. As far as sessile cells, the results showed the same antifungal activity, i.e., MIC values, for HA-Thy-25 and Thy after 24 and 48 h (250 and 500 μg/mL, Table S3). The MICs doubled after 2 days, with the highest values obtained for pristine HA at the two experimental times. For what concerns the biofilm inhibition, the BMIC values for **1**, **5a**, and HA are represented in Figure 3 (the numeric values in μg/mL are listed in Table S4).

**Figure 3:**
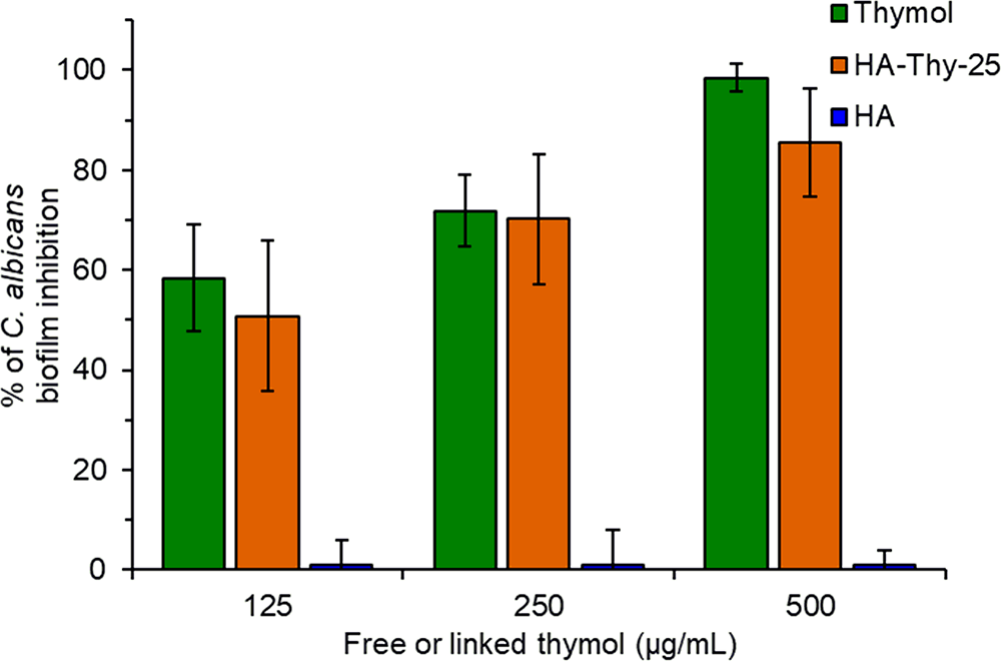
Inhibition of *C. albicans* ATCC 10231 biofilm formation in the presence of free thymol (green) and HA-Thy-25 (orange) after 48 h. At each point, HA concentration (mM) is equal to that of HA-Thy-25.

Hyaluronic acid does not display any inhibition against the *C. albicans* biofilm, having always a BMIC value of 0 μg/mL. As expected, bioactive molecule **1** gave a dose-dependent interference with the biofilm, with around 60% inhibition at 125 μg/mL (0.8 mM) that grows to 98% at 500 μg/mL (3.2 mM). Interestingly, the BMIC of HA-Thy-25 increases with the increase of concentration, varying from 50%, when linked-thymol is 0.8 mM, to 85%, when it is 3.2 mM. In this regard, it is worth noting that even if **1** possesses a massive antibiofilm activity, it is expressed in a concentration range in which the monoterpene is highly toxic versus fibroblast-like cells (concentration range between 0.8 and 3.2 mM, Figure 2). Conversely, conjugate **5a** maintains a certain level of interference against the *C. albicans* biofilm, albeit with good cell compatibility, comparable to that of virgin hyaluronic acid.

In conclusion, we report here the unprecedented synthesis of a HA-Thy bioconjugate with a degree of thymol functionalization of 25% and 50% relying on the introduction of a commercially available linker. The degree of functionalization was easily tuned by adjusting reagent ratios. The simplicity of the HA-Thy synthesis appears suitable for large-scale preparation. The successful esterification of thymol onto the HA backbone was evidenced by ^1^H NMR, ATR-FTIR, and UV characterization. By semiquantitative preliminary tests, the increase in conjugated-thymol water solubility was evidenced mainly for the derivative with 25% functionalization (HA-Thy-25). The latter was tested to assess its cytotoxic and antifungal effects against COS-7 line cells and *C. albicans*, respectively. Biological results clearly identified a concentration in which the material is completely noncytotoxic (similarly to free HA) and, simultaneously, shows an antibiofilm activity similar to free thymol. These results highlight the extreme efficiency of chemical conjugation in overcoming the insolubility of Thy in water and its toxicity, successfully pairing the properties of HA and Thy in a new biopolymer with promising applications as an antibiofilm formation material.

## Supporting Information Available

Materials and methods, synthetic and *in vitro* experimental procedures, ^1^H-NMR, ^13^C-NMR, ATR-FTIR spectra, and UV spectra for all presented compounds (PDF)
