# Supplementary material for: Thymol-Functionalized Hyaluronic Acid as Promising Preservative Biomaterial for the Inhibition of Candida albicans Biofilm Formation
Source: ACS Macro Lett. 2023 Jul 18;12(8):1079–84. doi: 10.1021/acsmacrolett.3c00208 (PMC10433527; doi:10.1021/acsmacrolett.3c00208)
Supplement: Supplementary file 1 — mz3c00208_si_001.pdf [file mz3c00208_si_001.pdf]

Supporting Information

Thymol-functionalized hyaluronic acid as promising preservative biomaterial for the inhibition of *Candida albicans* biofilm formation.

Elisa Sturabotti,[1]* Vyali Georgian Moldoveanu,[1] Alessandro Camilli,[1] Andrea Martinelli,[1] Giovanna Simonetti,[2] Alessio Valletta,[2] Ilaria Serangeli,[3] Alessandro Giustini,[3] Elena Miranda,[3] Luisa Maria Migneco,[1] Fabrizio Vetica,[1]* Francesca Leonelli[1]*

[1] *Department of Chemistry, Sapienza University of Rome, Piazzale Aldo Moro 5, 00185 Rome, Italy*

[2] *Department of Environmental Biology, Sapienza University of Rome, Piazzale Aldo Moro 5, 00185 Rome, Italy*

[3] *Department of Biology and Biotechnologies "Charles Darwin", Sapienza University of Rome, Piazzale Aldo Moro 5, 00185 Rome, Italy*

email: elisa.sturabotti@uniroma1.it, fabrizio.vetica@uniroma1.it, francesca.leonelli@uniroma1.it

Table of content

Materials and methods

Materials

NMR spectroscopy

Infrared spectroscopy

UV spectroscopy

Synthesis of Thy-ester (*4*)

Characterization of *4*

S1: ^{1}H NMR spectrum for compound ***4*** and its structure.

S2: ^{13}C NMR spectrum of compound ***4***.

S3: Comparison of ATR-FTIR spectra of compound ***4*** and pure thymol.

Synthesis of HA-TBA

Synthesis of HA-Thy-25 (*5a*)

Table S1: Molecular weights of all compounds.

S4: ATR-FTIR spectra of HA-Thy-25, HA-Thy-50 and Thy-ester compared to that of native HA.

S5: UV absorption spectra of pristine HA, thymol, HA-Thy-25 and HA-Thy-50 dissolved in water.

S6: ^{1}H NMR spectrum of HA-Thy-25 in D2O with peaks integrals and structure of compound ***5a***.

S7: ^{1}H NMR spectrum of HA-Thy-50 in D2O with peaks integrals.

S8: Comparison of ^{1}H NMR spectra in D2O of native HA, HA-Thy-25, HA-Thy-50 derivatives and Thy-ester.

Cell cultures and cellular viability MTT assay

Table S2. Concentrations of thymol, HA and HA-Thy-25 used in the MTT assay experiments.

***Candida albicans* strain and Culture Conditions**

Antifungal Susceptibility Testing (MIC)

Table S3. MIC measured for HA, Thy and HA-Thy-25 against *C. albicans* after 48 h of experiments.

Assays of Biofilm Inhibition (BMIC)

TableS4. Percentage of inhibition of *C. albicans* ATCC 10231 biofilm formation at 48 h for all the tested compounds.

Materials and methods

Materials

Hyaluronic acid was bought from Flower Tales Cosmetics (Milan, Italy) with an average MW of 1000 – 1500 kDa. Thymol (Thy), 4-bromobutyryl chloride, triethylamine (TEA), dichloromethane (DCM), tetrabutylammonium hydroxide (TBAOH), tetrabutylammonium iodide (TBAI), dimethyl sulfoxide (DMSO) and dialysis membrane (cut-off 14 kDa) were purchased by Sigma-Aldrich (Milan, Italy). HA was purified by extensive dialysis in water and then freeze dried. All other materials were used without any further purification.

NMR spectroscopy

Water soluble HA-Thy samples were dissolved in D_2O at r.t. (6 mg in 0.6 ml of D_2O). Thy-ester spectrum was recorded in $CDCl_3$. NMR analyses were performed on a 400 MHz Bruker Avance III spectrometer and spectra were processed using MestReNova 6.0.2 (Mestrelab Research SL). Third order polynomial fit was used to correct base line. Residual internal solvent was used as standard.

Infrared spectroscopy

Fourier-transform infrared spectroscopy (FTIR) was used to characterized HA-Thy derivatives. Spectra, before and after chemical modification, were acquired in attenuated total reflection (ATR) using a Nicolet 6700 (Thermo Fisher Scientific, Waltham, MA, USA) equipped with a Golden Gate single reflection diamond ATR accessory. All spectra were recorded in absorption mode with 200 scans/spectrum and at 4 cm^{-1} resolution in the region between 4000 – 650 cm^{-1}. The software Omnic 8.0 (Thermo Fisher Scientific Inc.) was used for the elaboration of spectra.

UV spectroscopy

UV spectra were recorded on HP DIODE ARRAY instruments between 190 and 820 nm, with 2 nm resolution. Water soluble samples were dissolved in Milli-Q water and analyzed in quartz cuvettes.

Synthesis of Thy-ester (*4*)

Thymol (1 mmol) and TEA (1.4 mmol) were dissolved at r.t. in dry DCM (10 ml) and cooled at 0 °C in an ice bath. After 10 min, 4-bromobutyryl chloride (1.4 mmol) was carefully added to the solution and stirred at 0 °C further for 1 h. Reaction was followed by TLC (hexane:diethyl ether=8:2) and after 3 h at r.t., the reaction was washed three times with water (3x50 ml), dried over Na_2SO_4, filtered and vacuum dried. Product was purified by column chromatography (silica flash from 10% v/v diethyl ether in hexane up to 20%) to obtain a pale yellow oil. Yield = 94% NMR and FTIR spectra in figure S1, S2 and S3, respectively.

Characterization of *4*

^{1}H NMR (400 MHz, $CDCl_3$) δ 7.24 – 6.74 (m, 3H, Ar), 3.56 (t, J = 6.4 Hz, 2H, **CH_2**Br), 2.96 (hept, J = 7.0 Hz, 1H, CH), 2.80 (t, J = 7.2 Hz, 2H, **CH_2**CO), 2.42 – 2.20 (m, 5H, CH_3 and CH_2**CH_2**CH_2), 1.20 (d, J = 7.0 Hz, 6H, 2xCH_3).

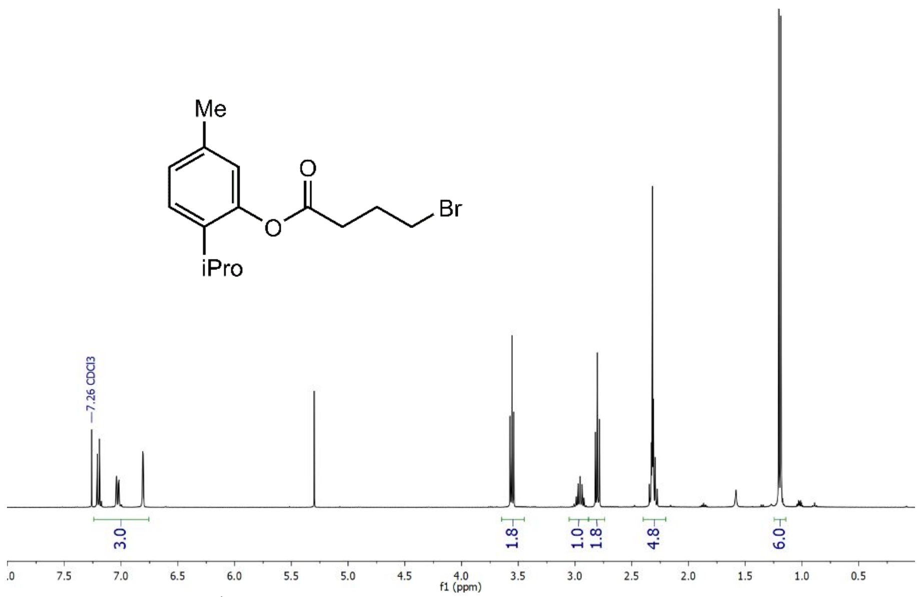

Figure S1. ^{1}H NMR spectrum in $CDCl_3$ for compound ***4*** and its structure.

^{13}C NMR (101 MHz, $CDCl_3$) δ 171.5, 147.9 137.1, 136.8, 127.4, 126.6, 122.8, 32.7, 32.6, 27.8, 27.3, 23.2, 21.0.

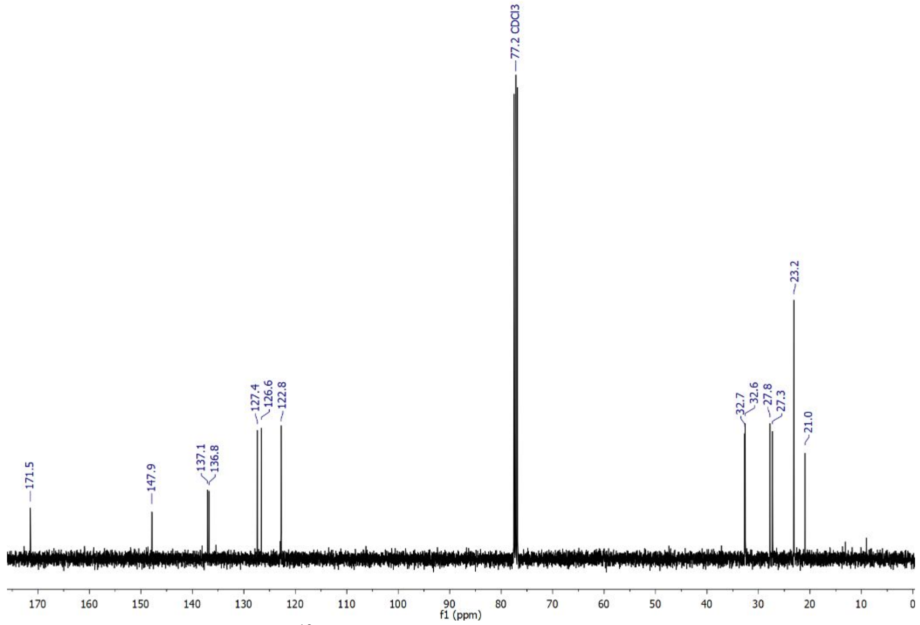

Figure S2. ^{13}C NMR spectrum of compound ***4*** in $CDCl_3$.

ATR-FTIR (oil): main peaks at 2960, 2920, 2870, 1750, 1620, 1500, 1150, 1120, 817 cm^{-1}.

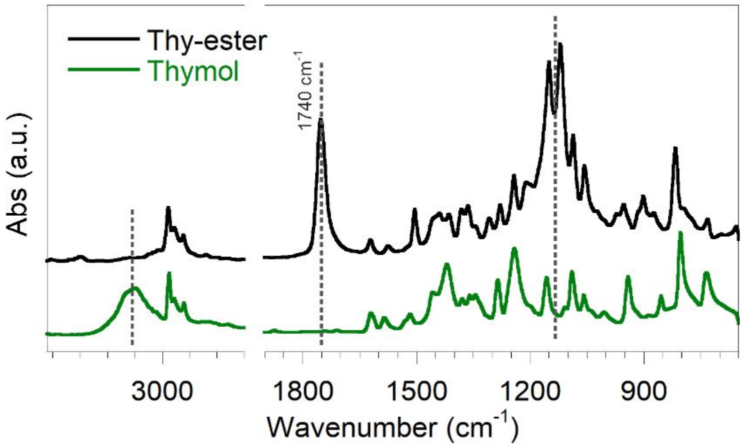

Figure S3. ATR-FTIR spectrum of compound ***4*** (green) compared to that of pure thymol (black). The formation of ester linkage is confirmed by the appearance of the C=O stretching band at 1740 cm^{-1}, together with that at 1100 cm^{-1} related to C-O-C stretching. Furthermore, the disappearance of ν OH band centered at 3180 cm^{-1} confirms the formation of ester moiety on the phenolic ring of thymol.

Synthesis of HA-TBA

HA-TBA was synthetized slightly modifying reference N° 25. Sodium hyaluronate (1 g, 2.5 mmol) was mixed with TBAOH (TBAOH in a 40% wt/v in water, 87 mmol), after proper activation with Dowex®50WX-8-400 resin (58 mmol). The mixture was stirred for 5 h at r.t., filtered and HA-TBA solution was purified by extensive dialysis against distilled water. Finally, HA-TBA salt was freeze dried and it was collected as a cotton-like material. TBA^+ ion presence was confirmed by intense stretching peaks of methyl and methylene groups between 3000 and 2880 cm^{-1} in the infrared spectrum of HA-TBA salt. Furthermore, proton spectrum in D_2O showed specific sets of signals assigned to the ammonium cation such as a triplet at 0.95 ppm for methyl groups and a sextuplet at 1.37, a quintuplet at 1.66 and a triplet at 3.20 ppm for the three methylene groups. Degree of substitution resulted to be close to 100% as indicated by ^{1}H NMR spectrum.

Synthesis of HA-Thy (*5*)

To a solution of HA-TBA (50 mg, 0.07 mmol) in DMSO (2.5 ml), Thy-ester (**4**) was added using two different reactants ratio (HA-TBA:Thy-ester=1:0.25 and HA-TBA:Thy-ester=1:0.50). TBAI salt was added as catalyst (0.02 mmol). Solutions were kept under stirring until TLC (Hex:Et=8:2) revealed consumption of **4**. Generally, 5 days of reaction were needed for its the complete consumption. After reaction time, the polymeric product was precipitated adding brine (1 ml) and ethanol (20 ml) to the mixture and stirred for additional 3 h. The HA precipitate was separated by centrifugation and purified by dialysis (cut-off 14 kDa) against Milli-Q water for 5 days. Extensive dialysis was necessary for the complete purification of HA derivatives from bromide anion, excess of NaCl or solvents. Finally, HA-Thy bioconjugate was frozen in liquid nitrogen, freeze dried and collected as a white cotton-like material.

Degree of thymol functionalization (TF%) was calculated from the ratio between aromatic signals of thymol between 7.5 and 6.7 ppm and methyl peak of HA unit at 2.0 ppm.

$$TF\ (\%) = \frac{A\ (Aromatic\ protons)^{Thymol}}{A(CH_3)^{HA}} * \ \%$$

Table S1. Molecular weights of all the tested compounds in the paper.

	**Compound**	**MW (g/mol)**
**1**	Thymol	150.22
**2**	Hyaluronic acid (sodium salt)	401.30
**3**	HA-Thy-25	450.38
**4**	HA-Thy-50	499.46

ATR-FTIR: 3360 (ν OH), 2900, 1745 (ν ester C=O), 1660 (ν amide I), 1605 (ν COONa), 1558 (ν amide II), 1400, 1375, 1311, 1150, 1080, 1042, 950, 900 cm^{-1}.

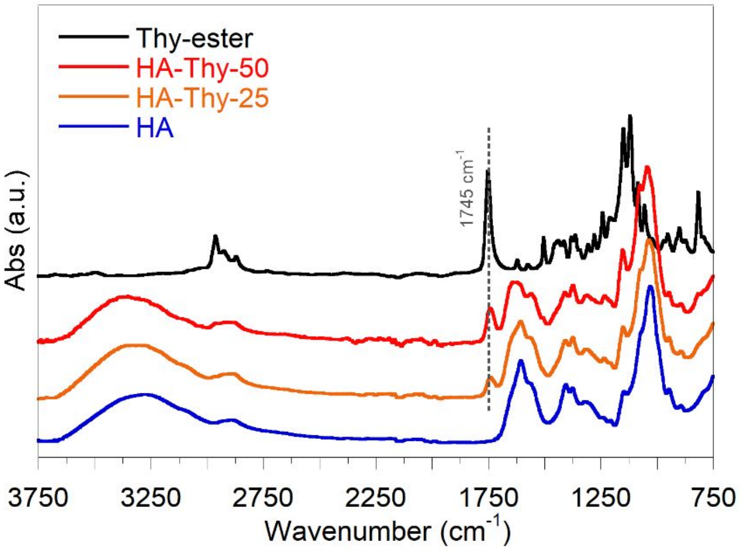

Figure S4. ATR-FTIR spectra of HA-Thy-25 (orange) and HA-Thy-50 (red) and Thy-ester (black) compared to that of native HA (black). The formation of a new ester bond by the linkage of thymol causes the increase of ester absorption band at 1745 cm^{-1} at the expense of COONa band at 1605cm^{-1}.

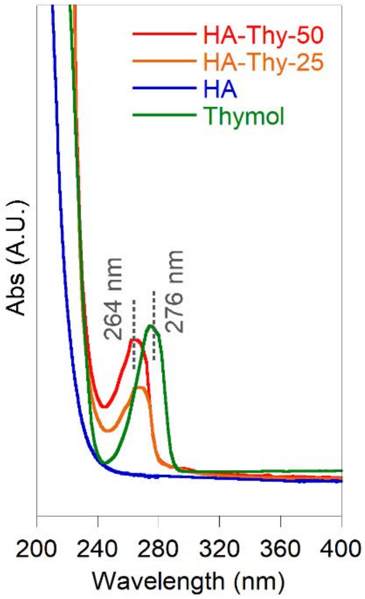

Figure S5. UV absorption spectra of pristine HA (blue), thymol (green), HA-Thy-25 (orange) and HA-Thy-50 (red) dissolved in water. The functionalization of HA with thymol causes the appearance of an absorption peak situated at 264 nm in the two HA derivatives. Free-phenolic thymol structure absorbs at 276 nm.

^{1}H NMR (400 MHz, D_2O): δ 7.5 – 6.7 (m, Ar-Thy-ester), 4.6 – 4.3 (d, HA anomeric protons), 4.1 – 3.1 (HA disaccharide unit), 2.9 (m, Thy-ester), 2.8 (s, Thy-ester), 2.3 (s, Thy-ester), 2.2 (s, Thy-ester), 2.0 (s, CH_3 HA), 1.2 (d, Thy-ester).

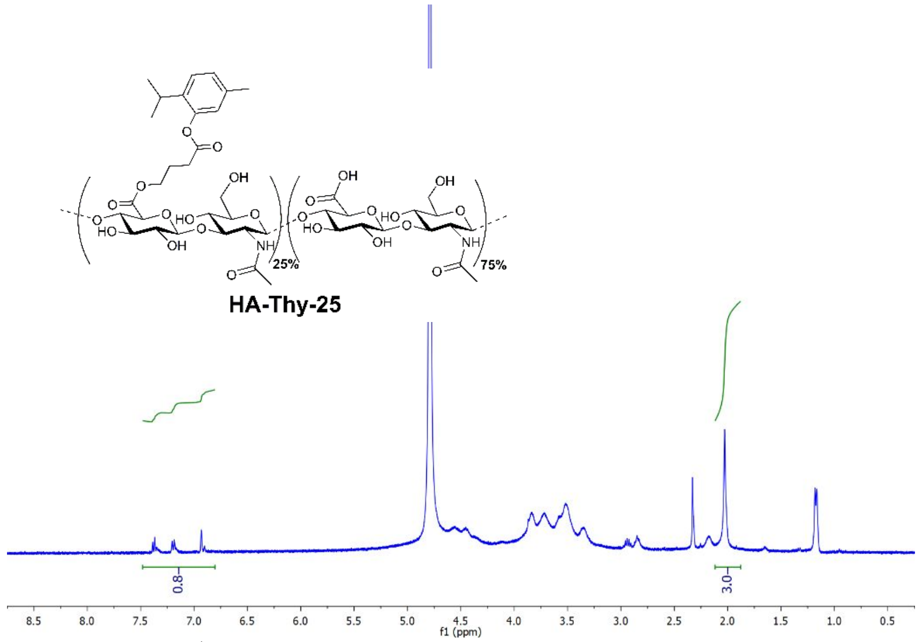

Figure S6. ^{1}H NMR spectrum HA-Thy-25 in D_2O and bioconjugate structure.

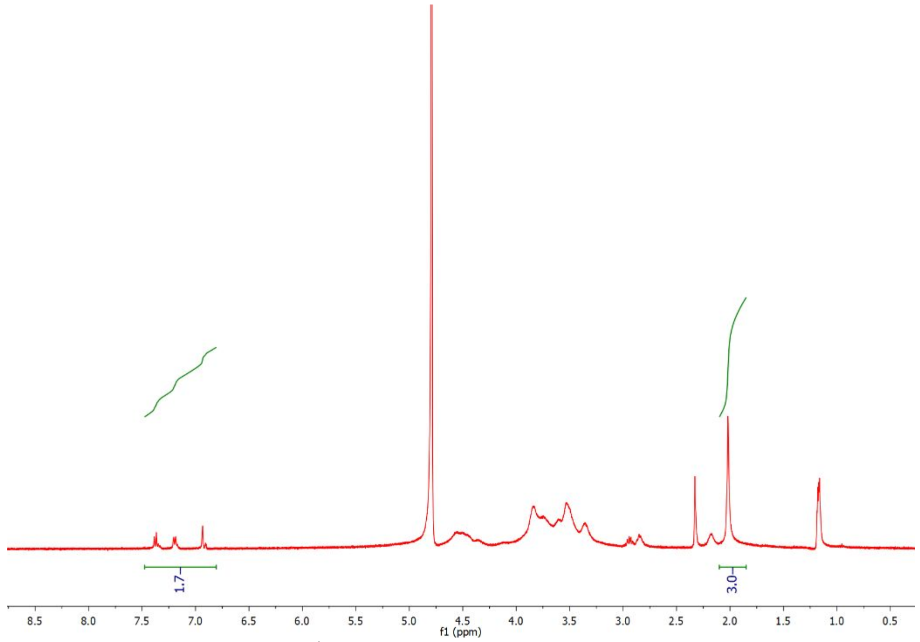

Figure S7. ^{1}H NMR spectrum of HA-Thy-50 in D_2O.

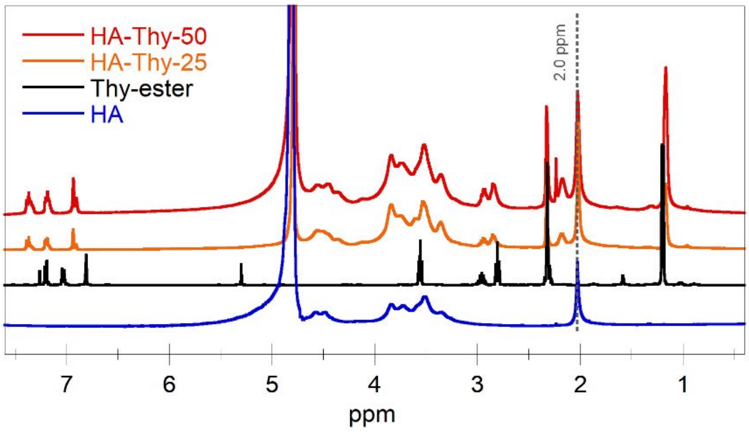

Figure S8. Comparison between ^{1}H NMR spectra in D_2O of native HA (blue), HA-Thy-25 (orange) and HA-Thy-50 (red) derivatives and of Thy-ester (black) in $CDCl_3$. Grafting of Thy-ester on HA backbone is evidenced by the appearance of peaks at c.a. 7.2, 2.9, 2.8, 2.3, 2.2 and 1.2 ppm in HA-Thy derivatives spectra.

Cell cultures and cellular viability MTT assay

COS-7 fibroblast cells (ECACC 87021302) were seeded at a density of 100,000 cells per well in a 24-well plate containing complete high glucose DMEM medium supplemented with 10% glutamine and 10% FBS. The compounds HA, HA-Thy-25, and free thymol were weighed under semi-sterile conditions and mechanically dissolved in Optimem Medium for 24 h at r.t. After 24 h, these solutions were added in the 24 well plate starting from the initial concentration of 12.8 mM for HA and HA-Thy-25, and 3.2 mM for free thymol, and then diluted 1:2 in Optimem Medium directly in well. Following a 48-hour incubation, cell viability was assessed using the MTT colorimetric assay (Abcam, ab211091), following the manufacturer's protocol. Briefly, the assay involved an incubation of 1.5 h at 37 °C, followed by measurement of the absorbance of the resulting solutions at 590 nm using a spectrophotometer (ThermoFisher).

Table S2. Concentrations of thymol, HA and HA-Thy-25 used in the MTT assay experiments.

	Thymol (mM)	HA (mM)	HA-Thy-25 (mM)
**1**	0.10	0.40	0.40
**2**	0.20	0.80	0.80
**3**	0.40	1.60	1.60
**4**	0.80	3.20	3.20
**5**	1.60	6.40	6.40
**6**	3.20	12.8	12.8

Candida albicans strain and culture conditions

In this study, *Candida albicans* ATCC 10231 (American Type Collection, Manassas, VA, USA) was utilized. *C. albicans* was grown for 24 h on Saboraurd Dextrose Agar (SDA) (Sigma-Aldrich, St. Louis, MO, USA). Five colonies of *C. albicans* were then collected with phosphate-buffered saline (PBS) and the inoculum was determined by spectrophotometric reading (Ultrospec™ 2100 pro) and was confirmed with the reading at the Bürker chamber.

Effect of Thymol, HA and HA-Thy-25 on *Candida albicans* planktonic cells.

The minimum inhibitory concentration (MIC) of thymol, HA-Thy and HA against *C. albicans* (ATCC 10231) was determined according to the standardized method (CLSI M27-A3 document; CLSI M27-S4). The final concentration of the inoculum was $1 \times 10^3 - 5 \times 10^3$ cells/mL. The compounds in RPMI were added to the wells. The concentrations of thymol ranged from 500 µg/mL to 0.977 µg/mL. The plates, incubated at 37 °C for 24 h and 48 h, were observed for growth inhibition compared to untreated growth controls. The minimum inhibitory concentration that caused growth inhibitions ≥ 50% (MIC) was determined. The antifungal activities are the result of three independent experiments performed in triplicate and reported as median. All experiments were carried out, in triplicate, at least three times on separate dates and on different HA-Thy-25 batches.

Table S3. Antifungal activity of thymol, HA-Thy-25 and HA against *Candida albicans* ATCC 10231.

Substances	*Candida albicans* ATCC10231	
	MIC 24 h Median (µg/mL)	MIC 48 h Median (µg/mL)
Thymol	250	500
HA-Thy-25	250	500
HA	500	1000
Amphotericin B	0.5	1
Fluconazole	1	128

Effect of Thymol, HA and HA-Thy-25 on *Candida albicans* biofilm formation

The anti-biofilm activity was assessed in 48-well plates as described previously (Simonetti G. et al. Molecules 24: 2070. 2019). *C. albicans* (ATCC 10231) was grown on SDA for 24 h. Five colonies were collected and counted with a hemocytometer. The inoculum concentration was 1.0×10^6 cells/mL in RPMI 1640 medium. The molecules were added to 48-well plates at the concentrations ranging from 125 µg/mL to 500 µg/mL. Then, the plates were incubated statically at 37 °C for 48 h. Biofilm mass was quantified with the crystal violet assay as previously reported by Ourabah, et al. (Journal of Herbal Medicine 20 (2020): 100319). Briefly, after biofilm formation, the medium was aspirated, and non-adherent cells were removed by washing with physiological salt solution. Biofilm was stained with crystal violet solution, then washed with sterile water to remove excess stain. Subsequently, biofilm was decolorized by the addition of 95% ethanol solution to each well. Finally, the solutions from each well were transferred to a new 96-well plate and their absorptions read at 570 nm in a microplate reader (Thermo Electron Type 355 MultiskanEX, Vantaa, Finland). Each experiment was executed at least three times, in triplicate, on separate days and on different fully characterized HA-Thy-25 batches. The inhibition was expressed as percentage and calculated using this formula:

$$Inhibition\ (\%) = 100 - \left(\frac{OD_{490}^{Sample\ well}}{OD_{490}^{Positive\ control}}\right) * 100$$

Absorbance values of the negative control (containing no cells) were subtracted from the values of the test wells to minimize background interference. The experiments were performed four times independently in triplicate and the results were expressed as mean ± standard deviation (SD).

Moreover, the minimum inhibitory concentration that caused the inhibition of biofilm formation ≥ 50% (BMIC) was determined.

TableS4. Percentage of inhibition of *Candida albicans* ATCC 10231 biofilm formation at 48 h. Amount values (µg/mL) correspond to the amount of free thymol or thymol in HA-Thy-25. HA concentration (mM) is equal to that of HA-Thy-25.

Substance	500 µg/mL (%)	Standard deviation (%)	250 µg/mL (%)	Standard deviation (%)	125 µg/mL (%)	Standard deviation (%)
Thymol	98	11	72	7	58	3
HA	0	5	0	7	0	3
HA-Thy-25	85	15	70	13	51	11
